# Metagenomic Analysis of Cecal Microbiome Identified Microbiota and Functional Capacities Associated with Feed Efficiency in Landrace Finishing Pigs

**DOI:** 10.3389/fmicb.2017.01546

**Published:** 2017-08-11

**Authors:** Zhen Tan, Ting Yang, Yuan Wang, Kai Xing, Fengxia Zhang, Xitong Zhao, Hong Ao, Shaokang Chen, Jianfeng Liu, Chuduan Wang

**Affiliations:** ^1^National Engineering Laboratory for Animal Breeding, MOA Key Laboratory of Animal Genetics and Breeding, Department of Animal Genetics and Breeding, China Agricultural University Beijing, China; ^2^The State Key Laboratory of Animal Nutrition, Institute of Animal Sciences, Chinese Academy of Agricultural Sciences Beijing, China; ^3^Beijing General Station of Animal Husbandry Beijing, China

**Keywords:** metagenomics, cecal microbiome, feed efficiency, feed conversion ratio, pigs

## Abstract

Feed efficiency (FE) appears to vary even within closely related pigs, and may be partly affected by the diversity in the composition and function of gut microbes. To investigate the components and functional differences of gut microbiota of low and high FE pigs, high throughput sequencing and *de novo* metagenomics were performed on pig cecal contents. Pigs were selected in pairs with low and high feed conversion ratio. The microorganisms of individuals with different FE were clustered according to diversity. The genus *Prevotella* was the most enriched in both groups, and the abundance of species *Prevotella* sp. *CAG:604* was significantly increased in low efficiency individuals compared to that in animals showing high efficiency. In contrast, other differential species, including lactic acid bacteria, were all enriched in the group with good feeding characteristics. Functional analysis based on the Kyoto Encyclopedia of Genes and Genomes databases demonstrated that differential genes for the metabolism of carbohydrates were most abundant in both groups, but pathways of pyruvate-related metabolism were more intense in pigs with higher FE. All these data indicated that the microbial environment was closely related to the growth traits of pigs, and regulating microbial composition could aid developing strategies to improve FE for pigs.

## Introduction

Genetics and the environment together shape the performance of domestic animals, and diet plays an extremely important role in production (Aggrey et al., 2010). A major way to reduce costs in the pig industry is to improve feed efficiency (FE), because feed accounts for more than 60% of the production cost (Jing et al., 2015). To enhance pig production, FE should be improved, and this can be measured using residual feed intake (RFI) or feed conversion ratio (FCR). The FCR is the amount of feed consumed per unit of body weight gain during a specified period, and is calculated as the feed intake divided by the weight gained. Thus, an animal with a high FCR is less efficient at converting feed into body mass than one with a low FCR. In previous studies, the heritability values for FCR ranged from 0.13 to 0.31 (Do et al., 2013).

Owing to the rapid development of metagenomic studies, the gut microbiota was identified as markedly influencing animal health and performance. Correlations have been determined between intestinal microbes and host. The scientific community’s understanding of intestinal microflora has improved. The gut microbiota improves the energy harvesting capacity (Turnbaugh et al., 2006). Changes in the gut physiology and gut microbial composition were reported to improve FE, together with genetic changes (Lumpkins et al., 2010). The microflora can have a negative impact on the host in various ways, including using excessive amounts of energy, and causing host energy diversion to the immune system by inducing inflammatory responses (Turnbaugh et al., 2006). Variation in the diversity of gut microbes has been associated with differences between different breeds of pigs (Yang et al., 2014), and various growth stages (Kim et al., 2015) or intestinal segments (Kim and Isaacson, 2015), as a result of different genetic and environmental factors. Some nutrients such as resistant starch (RS), which cannot be digested completely in the small intestine, need to be fermented by cecal and colonic microbes to produce short-chain fatty acids (SCFAs) (Knudsen et al., 2012). Feed conversion efficiency is closely related to the genetic diversity of the gut microbiota (Singh et al., 2012, 2014). Therefore, even for animals reared under the same environmental conditions, there is a clear link between the gut microbiota and pig productivity.

The gut microbiota was demonstrated to participate in the regulation of energy harvesting efficiency of the host, which was significantly associated with body weight gain (Looft et al., 2012, 2014; Kim et al., 2015; Kim and Isaacson, 2015). The microorganisms harbored in the gastrointestinal tract of animals have coevolved with their hosts (Ley et al., 2006). The study of community structure and functional capacity of gut microbiota was helpful for better understanding of the relationships between microbial function and host physiology and metabolism. Revealing the taxonomic composition and functional capacity of gut microbiota and their interaction with the host should facilitate understanding the roles they play in the host, and may improve pig production by increasing the component of FE associated with microorganisms. The study on the fecal microbiome in different fatness found that the cecal microbiome has the stronger ability to degrade xylan, pectin and cellulose (Yang et al., 2016) and taxon and functional capacity of fecal microbiota in low and high FCR broilers (Singh et al., 2014), there also has been not many researches on the functional capacity of gut microbial species associated with FE in animals, including pigs.

In this study, high throughput sequencing of metagenomes were undertaken to investigate differences between the microbial communities found in the cecal contents of female finishing Landrace pigs with high and low FE. We determined whether the different structural and functional characteristics of the bacterial populations between the two groups (high and low FE) were correlated with pig production performance. The putative microorganisms identified were associated with nutrient digestion and growth traits. This study may lead to an improved understanding of digestion in the large intestine, while providing novel insights into growth traits.

## Materials and Methods

### Animals and Tissues

In this trial, feed intake and body weight of 120 female Landrace pigs of 120–165 days of age were recorded using a Velos (Nedap Co., Ltd., Groenlo, Netherland) automated individual feeding system, which recorded each pig’s intake of feed and weight by recognizing the electronic ear mark during the feed eating per time. The FCR (FCR, feed intake divided by the weight gained) was then obtained. The pigs were provided the same commodity feed and clean water *ad libitum* throughout the experiment, and housed in an environmentally controlled room (10 pigs in each pen), provided by the Tianjin Ninghe Primary Pig Breeding Farm (Ninghe, China). All the experimental pigs were kept healthy and were provided a full range of feed lacking antibiotics and medicines. Pedigree information of all animals was available. The FCR in the high FE group (20 animals) was significantly different from that of the low FE group (20 animals) (**Supplementary Figure S1**). Two full-sib pairs and two half-sib pairs were selected, with each pair having opposite FCR phenotypes (Supplementary Table S1). We defined the low (Lce) group as individuals with low FE and high FCR values, and pigs with high FE and low FCR values were in the high (Hce) group.

The eight selected pigs were euthanized at 166 days of age, and digesta samples were collected from the cecum lumen of each pig within 20 min of euthanization. All samples were collected in sterile tubes and stored in liquid nitrogen as soon as possible until required for further analysis. All the methods were carried out in accordance with the approved guidelines (GB/T 17236–2008) from the Quality Supervision, Inspection, and Quarantine of the People’s Republic of China. All experimental protocols were approved by the Animal Welfare Committee of China Agricultural University (permit number: DK996).

### DNA Preparation and Sequencing

DNA was extracted and purified from cecal contents using QIAamp DNA Stool Mini Kit (Qiagen Ltd., Germany) following the manufacturer’ s instructions. The integrity of DNA was checked using 1% agarose gel electrophoresis, and the concentration of DNA was measured using a UV-Vis spectrophotometer (NanoDrop 2000c, United States).

Metagenomic DNA libraries were constructed with an insert size of 350 base pairs (bp) for each sample following the manufacturer’s instructions (Illumina) (Qin et al., 2010). The libraries for metagenomic analysis were sequenced on an Illumina HiSeq 2500 platform by an Illumina HiSeq - PE150 bp strategy.

All the sequencing data were deposited in the National Center for Biotechnology Information Short Read Archive under Accession no. SRP108960.

### Data Analysis

#### Quality Control

Illumina raw reads were treated to remove reads with low qualities, trim the read sequences, remove adaptor, and remove the host sequences. Specifically, reads with adaptor or more than 3 N bases were removed. The 3′ end of the read was trimmed and bases with low quality (Phred score < 20) were removed, as were reads with original length shorter than 50 bp. Host (pig) genomic DNA sequences were removed by SOAPaligner (v 2.21) (Li et al., 2008).

#### Taxonomy Annotation

The remaining clean reads were assembled by the SOAPdenovo software (v 1.05) (Li et al., 2008), mapping to microbial genomes in NCBI. The parameter is -m 4 -r 2 -m 100 -x 1000 (Qin et al., 2014). Then, the aligned reads were classified at Kingdom, Phylum, Class, Order, Family, Genus, and Species levels, and abundance was determined. The taxonomy profile was constructed at different levels. Principal component analysis (PCA) was used to determine whether there was any similarity between the composition of samples from the same condition. Box plots were generated with the coordinate values of each group, to judge if the first principal component and the second principal component had a significant effect on the sample distribution. Based on the non-parametric Kruskal–Wallis (KW) sum-rank test, LDA Effect Size (LEfSe) analysis was performed at the species level to determine the community or species that had a significant effect on the division of the samples (Segata et al., 2011).

#### Construction of Gene Set and Differential Gene Analysis

Using SOAPdenovo software (version 1.05), which is based on the De-Bruijn graph (Li et al., 2010), the filtered data were assembled by different sizes of Kmer (51, 55, 59, and 63). Scaffolds were broken into new scaftigs at their gaps. Meanwhile, we estimated the number of scaftigs ≥ 500bp, and selected assembly results with maximum N50 values (Qin et al., 2014).

MetaGeneMark (v 2.10) was employed for prediction of bacterial open reading frames (ORFs) (Noguchi et al., 2006). Gene sequence lengths less than 100 bp were filtered out, and the remaining sequences were translated into the corresponding amino acid sequences.

All predicted genes were clustered (identity > 95%, coverage > 90%) using CD-HIT^1^ (Li and Godzik, 2006). Selecting the longest gene sequence in each cluster and removing the redundant genes allowed construction of a non-redundant gene set.

Clean reads were compared to the non-redundant gene set by SOAPaligner (Li et al., 2008) matching the parameters described above. The abundance of each gene in the sample can be calculated based on the number of reads mapped to each gene. The sum of the abundance of each gene in one sample is 1, and the abundance is expressed as the relative abundance. According to the abundance of each gene, the differential genes were screened via the Wilcox rank-sum test, *p*-value was calculated (Qin et al., 2014).

#### Functional Annotation Analysis

The set of genes was aligned with the Kyoto Encyclopedia of Genes and Genomes (KEGG) gene database using BLAST (BLAST Version^2^ 2.2.28+) to obtain the KO annotation information from the KEGG database (Kanehisa et al., 2004). The differential genes between the groups by rank sum test were aligned with the KEGG database. Thus, the metabolic pathway information of each differential gene was obtained.

Gene sequences were used by BLAST to compare with the Carbohydrate-Active enzymes database (CAZy) to get the information on species source, the functional classification of EC, the gene and protein sequence, and the protein structure of CAZymes (Lombard et al., 2014), and with the Antibiotic Resistance Genes Database (ARDB) to obtain the type, quantity, and function information of antibiotic resistance genes (Liu and Pop, 2009).

## Results

### Sequencing of the Cecal Microbiota

The DNA of cecal digesta was extracted, fragmented, and sequenced by Illumina HiSeq PE-150, which generated a total of 62 Gbp of clean reads for eight samples, with an average sample size in the high group of 7.4 Gbp, and 8.1 Gbp in the low group. However, only 10 million reads were mapped to microbial genomes (Supplementary Table S2), because about three quarters of the reads originated from eukaryotic DNA, which belongs to feed residue. The microbial DNA could not be separated from eukaryotic DNA of the feed residue easily in experimental conditions, leading to a high proportion of contaminating reads. In addition, there were possibly many reads from microbes that exist in the environment but do not have genomic sequences present in the NCBI database (Hong et al., 2016).

### Taxonomic Composition of Cecal Microbiota

The taxonomic composition was calculated using metagenomics methods at the phylum and genus levels (**Supplementary Figures S2, S3**). A study by Looft et al. (2014) showed that, at the phylum level, Bacteroidetes was the dominant group in the cecum (Looft et al., 2014), and this result was close to the results from metagenomics analysis in this study. Approximately half of the bacteria were unclassified or accounted for extremely small percentages of all groups at the genus level.

**FIGURE 1:**
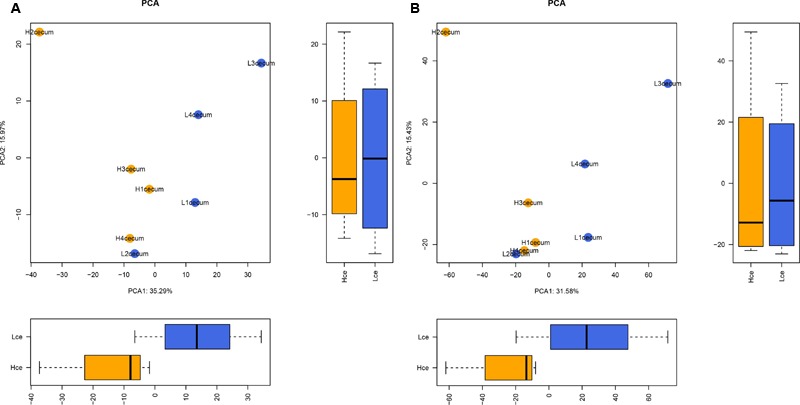
Microbial composition at the genus and species levels differs between cecal digest samples of high and low feed efficiency (FE). **(A)** Principal component analysis (PCA) of metagenomics based genera. **(B)** PCA of metagenomics based species. The boxes were tested by Wilcoxon test in the first dimension and second dimension between groups.

To evaluate the similarity of individuals in a group, and to elucidate whether the microbial composition had a connection with FE, PCA was performed based on the abundance profiling of microbes at the genus and species levels. **Figure 1A** shows that there were clear significant differences between the Hce and Lce groups in the first dimension (*P* = 0.029).

### Different Species of Cecal Microbiota between High and Low FE Groups

**FIGURE 2:**
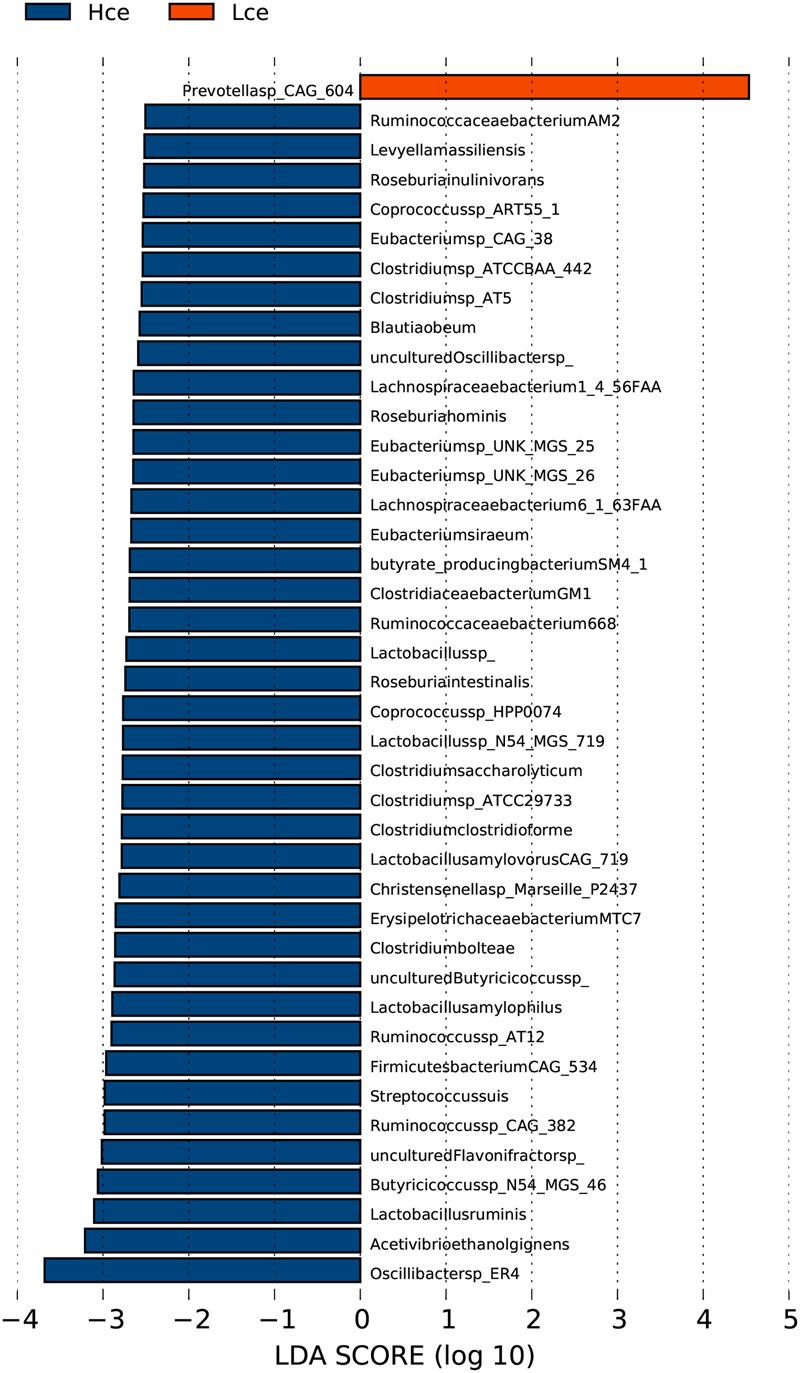
LDA Effect Size (LEfSe) analysis of cecal microbiota between high feed conversion ratio (FCR) and low groups at the species level. LDA (linear discriminant analysis) plot indicates biomarkers found by ranking accordingly to their effect size (2.0) of the species.

The PCA analysis revealed that differences in the organismal structure of cecal microbiota were present between the Hce and Lce groups (**Figure 1**). Differences in metagenomic taxonomic composition at the species level were identified. Over 300 species exhibited significant differences by the Wilcoxon test, and are shown in Supplementary Table S3. Interestingly, a comparable enrichment analysis of species between the two groups by linear discriminant analysis (LDA) plot revealed that some species were unique biomarkers to cecal microbes of the high or low FE group (**Figure 2**). The LDA Effect Size (LEfSe) based on non-parametric Kruskal–Wallis (KW) sum-rank test results showed only one species, *Prevotella* sp. *CAG:604*, had a significant effect, and could be considered as a potential biomarker for the Lce group. The remainder belonged to the Hce group, and *Oscillibacter* sp*_ER4* had the highest LDA score. *Oscillibacter* is known as a probiotic and producer of anti-inflammatory metabolites (Li et al., 2016). These biomarkers associated with nutrient metabolism and disease prevention showed significantly different abundance, consistent with the better overall health of the Hce group compared with that of the Lce group.

**FIGURE 3:**
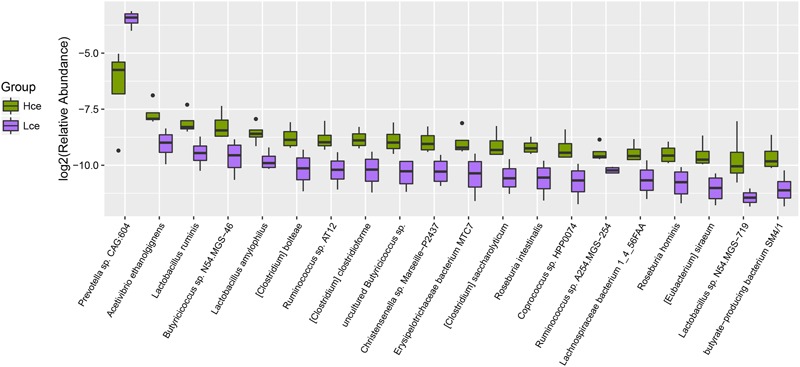
Boxplot for top 20 differentially enriched species between the Hce and Lce groups. Hce, cecal microbiota of high FE group. Lce, cecal microbiota of low FE group.

By mapping to the microbial genome, metagenomics species (MGS) were identified in both groups, with 314 species showing significant abundance differences (by Wilcoxon rank-sum tests) (Supplementary Table S3). From taxonomic characterization of the MGS, the top 20 different species showing enrichment are plotted in **Figure 3**. Nineteen of the top twenty species that differed were more enriched in the Hce group. *Prevotella* sp. *CAG:604* was the only single species concentrated more in the low group compared to that in the high group. *Prevotella* sp. *CAG:604* contains some genes that encode proteins involved in nutrient and energy metabolism, such as BN731_01873, ychF, gpmI, queF, speA, fmt, etc. There were three MGS of *Lactobacillus* (*Lactobacillus ruminis, L. amylophilus*, and *Lactobacillus* sp. *N54.MSG-719*) in the nineteen species enriched in the Hce group, with *Lactobacillus* spp. often considered as probiotics.

**FIGURE 4:**
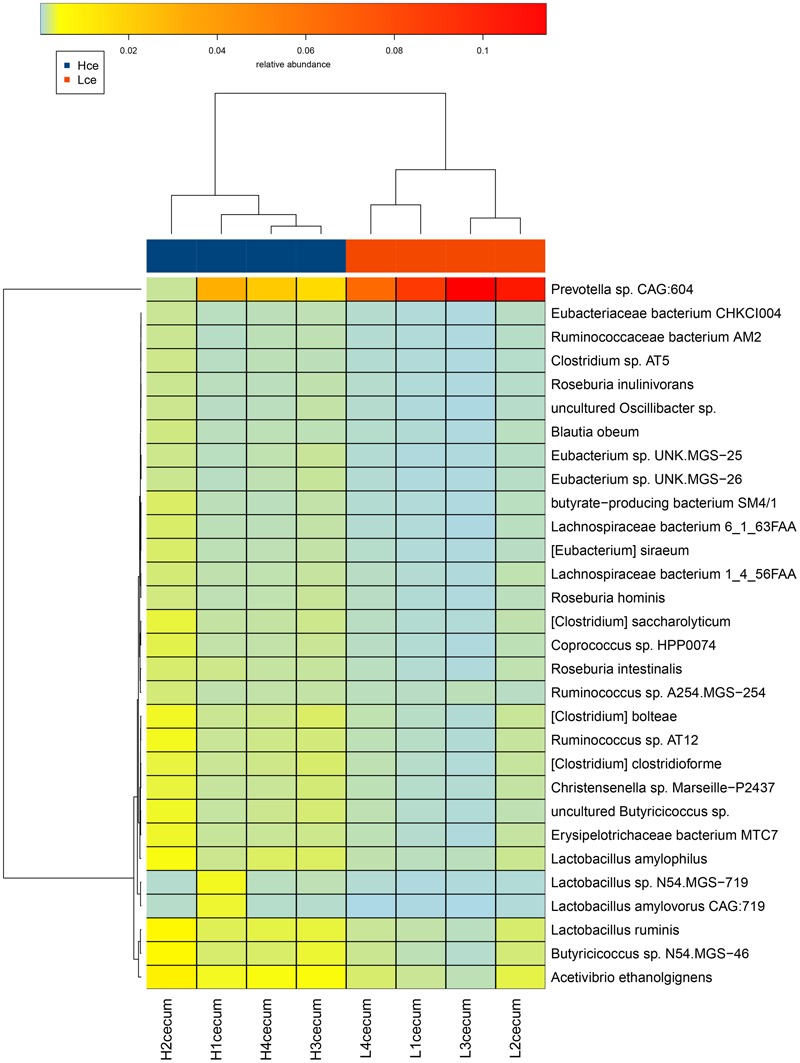
Heatmap cluster analysis of species based on differentially abundant cecal microbiota between the high and low FE groups. The relative levels of abundance are depicted visually from green to red; red represents the highest abundance, whereas green represents the lowest level of abundance. Vertical clustering indicates the similarity in expression between all species in different samples. Horizontal clustering indicates the similarity in abundance of the species of each sample.

A heatmap plot (**Figure 4**) shows when the major different species were clustered based on species similarity and relative abundance, *Prevotella* sp. *CAG:604* was clustered to a unique branch compared to other species. These results indicated that the species *Prevotella* sp. *CAG:604* might be a potential biomarker for distinguishing between the cecum microbiota of the high and low FE groups.

### Comparison of Functionality of the Cecal Microbiome between High and Low FE Groups

The functional classification results could help to clarify the metabolic differences between groups. The different aspects between high and low FCR phenotypes may possibly indicate the microbes that affect nutrient metabolism.

The different predicted genes are listed in Supplementary Table S4, with more than forty thousand (48221) assembled sequences showing significant differences by Wilcox rank-sum test with a *P*-value < 0.05 (Qin et al., 2014). To elucidate the distribution of samples based on different genes, PCA was performed for the eight samples (**Supplementary Figure S4**). The composition of predicted genes displayed visible differences between the groups.

The different genes were aligned to the CAZy, and categorized into seven CAZy types (**Supplementary Figure S5**) (Lombard et al., 2014). Glycoside hydrolases were enriched most in both high and low groups, and next were the glycosyl transferases.

Many coexisting microbes compete for nutrients in the cecal fluid, and some microbes may produce antibiotics or toxins to inhibit the growth of others. Analyzing genes by the ARDB can allow annotation of the abundance and the strength of antibiotic resistance genes (Hong et al., 2016). The heatmap of **Supplementary Figure S6** shows that from the abundant AR types after *z*-score processing, the type “MacAB” was the most aligned in both groups. However, the AR types clustered by samples were separated into two major areas. This suggested that the microbe-contributed AR genes were different between the two groups, and regulating the microbial composition might affect the growth of undesirable microbes in response to certain antibiotics.

**FIGURE 5:**
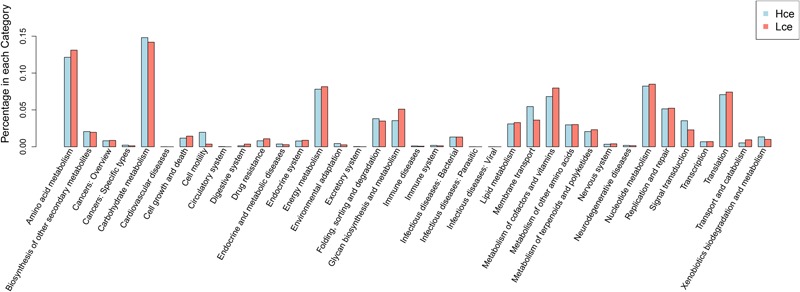
Distribution of category of differential genes by KEGG. KEGG, Kyoto Encyclopedia of Genes and Genomes; Hce, cecal predicted genes of high FE group. Lce, cecal predicted genes of low FE group.

Differential genes were dominated by the carbohydrate metabolism category in the clustering base subsystem of KEGG pathways, as expected, in **Figure 5**, where the differential genes between the groups by rank sum test were aligned with the KEGG database. The metabolic pathway information of each differential gene was obtained. Amino acid metabolism, energy metabolism, nucleotide metabolism, metabolism of cofactors and vitamins, and transcription were represented in relatively high abundance in both groups.

The differential pathways were then investigated (Supplementary Table S5), and 11 pathways were significantly different with *p* < 0.05. Apart from nitrogen metabolism and other glycan degradation, nine of the eleven pathways showed higher expression in the Hce group: ATP-binding cassette (ABC) transporters, bacterial chemotaxis, D-Arginine and D-ornithine metabolism, flagellar assembly, lysine degradation, phenylalanine metabolism, sulfur relay system, synthesis and degradation of ketone bodies, and two-component system. Bacterial chemotaxis and flagellar assembly are closely related, affected microbiological performance and development. ABC transporters play roles in the import of essential nutrients, the export of toxic molecules, and also mediate the transport of many other physiological substrates (Davidson et al., 2008). The differential pathways categorized as synthesis and degradation of ketone bodies, sulfur relay system, lysine degradation, other glycan degradation, D-Arginine and D-ornithine metabolism, and phenylalanine metabolism were classified to nutrient metabolism. The higher abundance of these pathways indicted that the positive activities of some nutrient metabolism pathways in Landrace pigs may be involved in faster growth.

In addition, some differential genes in the Hce group that were related to ABC transporters, lysine degradation, and phenylalanine metabolism could be mapped to *Desulfovibrio piger*, which is considered to be an intestinal sulfate-reducing bacterium (Kushkevych, 2015) expressing pyruvate-ferredoxin oxidoreductase. *Lactobacillus* was matched to the two-component system in the Hce group, but *Prevotella* matched in the Lce group. *Prevotella* also matched nitrogen metabolism and other glycan degradation, two pathways that were enriched in the Lce group.

**FIGURE 6:**
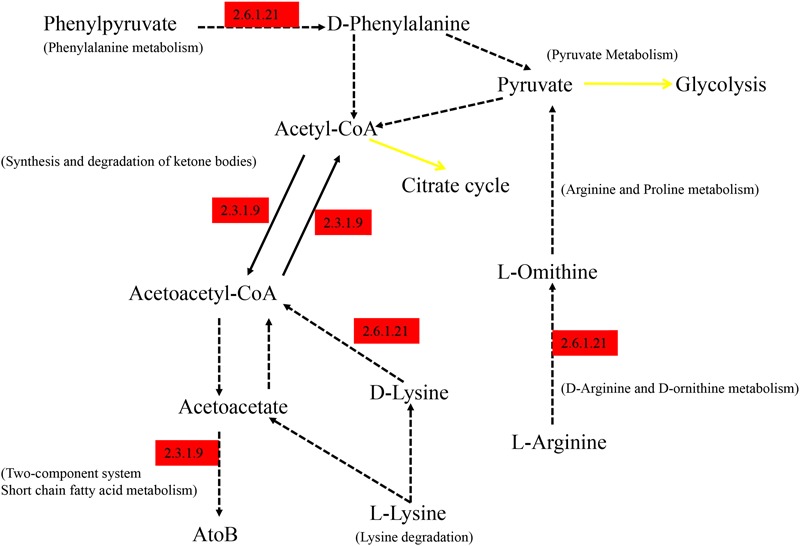
Summarized KEGG pathways of genes significantly abundant in Hce group comparing to Lce. Hce, cecal predicted genes of high FE group. Lce, cecal predicted genes of low FE group. Genes significantly abundant in the Hce are annotated in red; the black solid lines correspond to only one reaction on KEGG pathway; the yellow solid lines indicate a link to another map; the dotted lines indicate more than one reaction.

As shown in **Figure 6**, the differential genes were mapped to KEGG, the significantly abundant pathways differing between groups were obtained, and the majority of the pathways were interrelated and connected with degradation of nutrients. All the pathway information of **Figure 6** is annotated at the KEGG website. The EC 2.6.1.21 (D-amino-acid transaminase) enriched in Hce is responsible for degradation of amino acids. EC 2.3.1.9 is defined as acetyl-CoA *C*-acetyltransferase, which is involved in many pathways of degradation and metabolism.

## Discussion

To reduce false-positive results caused by genetic background noise and the number of replicates (Xing et al., 2016), two full-sib pairs and two half-sib pairs of pigs with opposing FCR phenotypes were used. Previous studies on broilers involving feed conversion efficiency trials have revealed that fecal bacteria are linked to body weight gain (Singh et al., 2014). The compositions and functional annotations of cecal microbiota were characterized for pigs with divergent FE phenotypes during the finishing period here. We searched for the presence of unique genera and/or genes potentially associated with digestion and absorption of nutrients.

The number of mapped reads was a little smaller, but excluding host DNA (average about 2%) and eukaryotic DNA of the feed residue (appropriate 75%), the proportion of mapped reads will be up to 20%, besides that the number of contigs was normal by the reference (Xiao et al., 2016). While the assembled contig length was 155.7 M ± 40.5 M, and total gene number was 999,791 ± 255,664 in Xiao et al found. The assembled contig length was 143.7 M, and total gene number was 650,604 in average in my study.

Firmicutes and Bacteroidetes were the most abundant phyla in cecal microbiota of piglets in both groups, accounting for more than 80% of the bacterial community, consistent with previous studies (Pedersen et al., 2013; Kim and Isaacson, 2015). The predominant three genera in the cecum of both groups were *Prevotella, Bacteroides*, and *Lactobacillus*. The Lce group had a higher proportion of *Prevotella* than the Hce group (**Supplementary Figure S3**). A higher abundance of *Prevotella* is probably related to the presence of fructo-oligosaccharides and starch in the lower intestine (Metzler-Zebeli et al., 2013). *Prevotella* was also considered to be a polysaccharide-degrading bacterial genus, associated with the ability to degrade mucin and plant-based carbohydrates (Lamendella et al., 2011; Patel et al., 2014).

Previous reports showed that *Lactobacillus* was involved in the metabolism of bile acids, which is related to obesity and metabolic syndrome, and the metabolism of phenolic, benzoyl, and phenyl derivatives, which is associated with weight loss (Nicholson et al., 2012). The different abundance of *Lactobacillus* might cause the diversity of energy metabolites in the high and low FE groups. *Lactobacillus*, a member of the lactic acid bacteria (LAB), is commonly used as a probiotic. Some species of *Lactobacillus* help to shape the composition of the gut microbiota by producing antimicrobial bacteriocins (Kim and Isaacson, 2015). Bacteroides fermented various sugar derivatives from plant material and have some benefits for the host, and members of Bacteroides affect the lean or obese phenotype in humans (Ridaura et al., 2013). Several species of *Lactobacillus* are LAB that are used as probiotics, which convert carbohydrates to lactic acid in homofermentation or heterofermentation, or to acetic acid in heterofermentation. The acidified environment produced by these two acids can inhibit the growth of other microbes (Sami et al., 1997).

The other top different MGS were also correlated with conversion and metabolism: *Acetivibrio ethanolgignens* could not degrade cellulose, but can produce ethanol; *Butyricicoccus* sp. *N54.MGS-46* may be a butyrate-producing microbe (Takada et al., 2016); and *Roseburia*, Lachnospiraceae and *butyrate-producing bacterium SM4/1* were also regarded as butyrate-producing bacteria (Duncan et al., 2002; Meehan and Beiko, 2014). Other studies showed that many species belonging to the family Erysipelotrichaceae were enriched in diet-induced obese animals (Turnbaugh et al., 2008). The more vigorous bacteria produced volatile fatty acids and their derivatives in the Hce group compared to that in the Lce group, suggesting that the digestion of the Hce group is more effective than that that of the Lce group and consistent with high FE.

From heatmaps and PCA plots, intra-group samples could be separated from inter-group ones. The *Prevotella* sp. *CAG:604* enriched in the Lce group was clustered away from other species enriched in the Hce group. The differential genes between the groups were analyzed with the KEGG gene database, and metabolic pathway information of differential genes was obtained. Carbohydrate metabolism was the metabolic pathway most enriched in both groups of differential genes. The non-digestible carbohydrates in the small intestine, including cellulose, xylan, and resistant starch, are fermented in the large intestine by microbiota to yield energy for microbial growth, and the end products are volatile fatty acids and their derivatives (Tremaroli and Backhed, 2012).

Carbohydrate metabolism was observed in both groups but involved significantly different genes. CAZymes in assembled different genes were screened for potential complex carbohydrate degradation (**Supplementary Figure S5**). Glycoside hydrolases (GHs) were enriched in both groups, indicating that the majority were related to carbohydrate hydrolysis. However, they were not the same genes in the two groups. Moreover, this result illustrated the pathways of carbohydrate metabolism might be variant between the Hce and Lce groups. From **Supplementary Figure S5**, more genes were concentrated in the Lce group, inconsistent with results from other studies that higher CAZy enzymes were associated with better digestive capacity of pigs (Ramayo-Caldas et al., 2016). When the results were clustered by differences in genes, rather than pathways, they differed.

The predicted genes were specifically annotated using the ARDB. Highly abundant annotations listed in a heatmap (**Supplementary Figure S6**) showed that the individuals of two groups could be grouped into two categories based on antibiotic resistance genes, with the concentrations in different categories. AR gene analysis suggested that diversity existed between individuals with various growth phenotypes. Suppressing the growth of undesirable microbes by regulating microbial composition might be helpful to promote healthy growth of animals (Hong et al., 2016). Health programs could possibly be developed to improve the growth efficiency of pigs by targeting different levels of the specific resistance gene in individuals with different FE.

The KEGG results were consistent with previous reports. The Hce group harbored a relatively high abundance of genes associated with pyruvate- and butyrate-related metabolism (**Figure 6**), part of carbohydrate metabolism, and one of the main sources of SCFAs that were later absorbed by the host (Krishnan et al., 2015). By mapping the differential genes in different pathways to the bacterial genome, *D. piger* was related to ABC transporters, lysine degradation, and phenylalanine metabolism, *Lactobacillus* matched to the two-component system in the Hce group, and *Prevotella* was related to nitrogen metabolism, other glycan degradation and the two-component system in the Lce group. These results were consistent with higher abundance of *Lactobacillus* in the Hce group and *Prevotella* in the Lce group.

Moreover, these differential pathways could be installed in series, and the EC 2.3.1.9 and EC 2.6.1.21 participating in these metabolic pathways were highly expressed in the Hce group. EC 2.3.1.9 (Acetoacetyl-CoA thiolase) catalyzes the cleavage of acetoacetyl-CoA into acetyl-CoA and its reverse reaction (Fox et al., 2014), and EC 2.6.1.21 (D-Amino acid aminotransferase) catalyzes the inter-conversion between various D-amino acids and alpha-keto acids (Fuchikami et al., 1998). They are responsible for fatty acid degradation and potentially produce medium chain fatty acids for energy supplementation, helping growth of the host.

The pathways of bacterial chemotaxis and flagellar assembly were also increased in the Hce group, suggesting a better growth environment for microorganisms in the Hce group than in the Lce group. These metabolic differences, and especially resulting compounds, possibly influence both microbial composition and growth of the host.

## Conclusion

In summary, there were differences in the cecal microbiota of individuals with different FE. Microorganisms that differed in abundance were mainly related to carbohydrate metabolism and may affect the growth of the host. The cecum of individuals with high FE contained more potential probiotics such as some species of *Lactobacillus*. Functional analysis revealed that the differentially expressed genes affect the host’s energy absorption mainly through the pathway of pyruvate-related metabolism. Taken together, these results indicated that the microbial environment was closely related to the growth traits of pigs, and regulation of microorganism composition could be applied to the pig production industry.

## Author Contributions

JL, CW, and ZT planned the project and designed the experiments. ZT conducted the experiments and carried out the data analysis with help from TY, KX, and YW. ZT, YW, KX, FZ, XZ, HA, and SC contributed reagents preparation and samples collection. ZT wrote the manuscript, which was critically reviewed by JL and CW.

## Conflict of Interest Statement

The authors declare that the research was conducted in the absence of any commercial or financial relationships that could be construed as a potential conflict of interest.

## Footnotes

1 http://www.bioinformatics.org/cd-hit/

2 http://blast.ncbi.nlm.nih.gov/Blast.cgi

## Supplementary Material

FIGURE S1 Feed Conversion Ratio (FCR) calculated in high and low groups. Significant difference was tested of 20 individuals at the ends of the high and low FCR, respectively, by one way variance analysis.

FIGURE S2 Taxonomic composition calculated by different methods at the phylum level based on metagenomics.

FIGURE S3 Taxonomic composition calculated by different methods at the genus level based on metagenomics.

FIGURE S4 principal component analysis (PCA) for significant different predicted genes between the Hce and Lce groups. Hce, cecal predicted genes of high FE group. Lce, cecal predicted genes of low FE group.

FIGURE S5 Number of different abundant genes clustered by CAZy in Hce and Lce groups. CAZy, Carbohydrate-Active Enzymes Database; Hce, cecal predicted genes of high FE group; Lce, cecal predicted genes of low FE group.

FIGURE S6 Heatmap diagram showing the abundance of antibiotic resistant genes differs between the cecal microbiota of high and low FE groups. FE, feed efficiency. Homogenization control of row by *z*-score.

## References

[B1] Aggrey S. E. Karnuah A. B. Sebastian B. Anthony N. B. (2010). Genetic properties of feed efficiency parameters in meat-type chickens. *Genet. Sel. Evol.* 42:25 10.1186/1297-9686-42-25 PMC2901204 20584334

[B2] Davidson A. L. Dassa E. Orelle C. Chen J. (2008). Structure, function, and evolution of bacterial ATP-binding cassette systems. *Microbiol. Mol. Biol. Rev.* 72 317–364. 10.1128/MMBR.00031-07 18535149 PMC2415747

[B3] Do D. N. Strathe A. B. Jensen J. Mark T. Kadarmideen H. N. (2013). Genetic parameters for different measures of feed efficiency and related traits in boars of three pig breeds. *J. Anim. Sci.* 91 4069–4079. 10.2527/jas.2012-6197 23825329

[B4] Duncan S. H. Hold G. L. Barcenilla A. Stewart C. S. Flint H. J. (2002). *Roseburia intestinalis* sp nov., a novel saccharolytic, butyrate-producing bacterium from human faeces. *Int. J. Syst. Evol. Microbiol.* 52 1615–1620. 10.1099/ijs.0.02143-0 12361264

[B5] Fox A. R. Soto G. Mozzicafreddo M. Garcia A. N. Cuccioloni M. Angeletti M. (2014). Understanding the function of bacterial and eukaryotic thiolases II by integrating evolutionary and functional approaches. *Gene* 533 5–10. 10.1016/j.gene.2013.09.096 24120621

[B6] Fuchikami Y. Yoshimura T. Gutierrez A. Soda K. Esaki N. (1998). Construction and properties of a fragmentary D-amino acid aminotransferase. *J. Biochem.* 124 905–910. 9792912 10.1093/oxfordjournals.jbchem.a022206

[B7] Hong X. Chen J. Liu L. Wu H. Tan H. Xie G. (2016). Metagenomic sequencing reveals the relationship between microbiota composition and quality of Chinese Rice Wine. *Sci. Rep.* 6:26621 10.1038/srep26621 PMC4886530 27241862

[B8] Jing L. Hou Y. Wu H. Miao Y. Li X. Cao J. (2015). Transcriptome analysis of mRNA and miRNA in skeletal muscle indicates an important network for differential Residual Feed Intake in pigs. *Sci. Rep.* 5:11953 10.1038/srep11953 PMC4493709 26150313

[B9] Kanehisa M. Goto S. Kawashima S. Okuno Y. Hattori M. (2004). The KEGG resource for deciphering the genome. *Nucleic Acids Res.* 32 D277–D280. 10.1093/nar/gkh063 14681412 PMC308797

[B10] Kim H. B. Isaacson R. E. (2015). The pig gut microbial diversity: understanding the pig gut microbial ecology through the next generation high throughput sequencing. *Vet. Microbiol.* 177 242–251. 10.1016/j.vetmic.2015.03.014 25843944

[B11] Kim J. Nguyen S. G. Guevarra R. B. Lee I. Unno T. (2015). Analysis of swine fecal microbiota at various growth stages. *Arch. Microbiol.* 197 753–759. 10.1007/s00203-015-1108-1 25832348

[B12] Knudsen K. E. B. Hedemann M. S. Laerke H. N. (2012). The role of carbohydrates in intestinal health of pigs. *Anim. Feed Sci. Technol.* 173 41–53. 10.1016/j.anifeedsci.2011.12.020

[B13] Krishnan S. Alden N. Lee K. (2015). Pathways and functions of gut microbiota metabolism impacting host physiology. *Curr. Opin. Biotechnol.* 36 137–145. 10.1016/j.copbio.2015.08.015 26340103 PMC4688195

[B14] Kushkevych I. V. (2015). Kinetic properties of pyruvate ferredoxin oxidoreductase of intestinal sulfate-reducing bacteria *Desulfovibrio piger* Vib-7 and *Desulfomicrobium* sp Rod-9. *Pol. J. Microbiol.* 64 107–114. 26373169

[B15] Lamendella R. Domingo J. W. Ghosh S. Martinson J. Oerther D. B. (2011). Comparative fecal metagenomics unveils unique functional capacity of the swine gut. *BMC Microbiol.* 11:103 10.1186/1471-2180-11-103 PMC3123192 21575148

[B16] Ley R. E. Peterson D. A. Gordon J. I. (2006). Ecological and evolutionary forces shaping microbial diversity in the human intestine. *Cell* 124 837–848. 10.1016/j.cell.2006.02.017 16497592

[B17] Li J. Sung C. Y. J. Lee N. Ni Y. Pihlajamaki J. Panagiotou G. (2016). Probiotics modulated gut microbiota suppresses hepatocellular carcinoma growth in mice. *Proc. Natl. Acad. Sci. U.S.A.* 113 E1306–E1315. 10.1073/pnas.1518189113 26884164 PMC4780612

[B18] Li R. Li Y. Kristiansen K. Wang J. (2008). SOAP: short oligonucleotide alignment program. *Bioinformatics* 24 713–714. 10.1093/bioinformatics/btn025 18227114

[B19] Li R. Zhu H. Ruan J. Qian W. Fang X. Shi Z. (2010). *De novo* assembly of human genomes with massively parallel short read sequencing. *Genome Res.* 20 265–272. 10.1101/gr.097261.109 20019144 PMC2813482

[B20] Li W. Godzik A. (2006). Cd-hit: a fast program for clustering and comparing large sets of protein or nucleotide sequences. *Bioinformatics* 22 1658–1659. 10.1093/bioinformatics/btl158 16731699

[B21] Liu B. Pop M. (2009). ARDB-antibiotic resistance genes database. *Nucleic Acids Res.* 37 D443–D447. 10.1093/nar/gkn656 18832362 PMC2686595

[B22] Lombard V. Golaconda Ramulu H. Drula E. Coutinho P. M. Henrissat B. (2014). The carbohydrate-active enzymes database (CAZy) in 2013. *Nucleic Acids Res.* 42 D490–D495. 10.1093/nar/gkt1178 24270786 PMC3965031

[B23] Looft T. Allen H. K. Cantarel B. L. Levine U. Y. Bayles D. O. Alt D. P. (2014). Bacteria, phages and pigs: the effects of in-feed antibiotics on the microbiome at different gut locations. *ISME J.* 8 1566–1576. 10.1038/ismej.2014.12 24522263 PMC4817603

[B24] Looft T. Johnson T. A. Allen H. K. Bayles D. O. Alt D. P. Stedtfeld R. D. (2012). In-feed antibiotic effects on the swine intestinal microbiome. *Proc. Natl. Acad. Sci. U.S.A.* 109 1691–1696. 10.1073/pnas.1120238109 22307632 PMC3277147

[B25] Lumpkins B. S. Batal A. B. Lee M. D. (2010). Evaluation of the bacterial community and intestinal development of different genetic lines of chickens. *Poult. Sci.* 89 1614–1621. 10.3382/ps.2010-00747 20634515

[B26] Meehan C. J. Beiko R. G. (2014). A phylogenomic view of ecological specialization in the lachnospiraceae, a family of digestive tract-associated bacteria. *Genome Biol. Evol.* 6 703–713. 10.1093/gbe/evu050 24625961 PMC3971600

[B27] Metzler-Zebeli B. U. Schmitz-Esser S. Klevenhusen F. Podstatzky-Lichtenstein L. Wagner M. Zebeli Q. (2013). Grain-rich diets differently alter ruminal and colonic abundance of microbial populations and lipopolysaccharide in goats. *Anaerobe* 20 65–73. 10.1016/j.anaerobe.2013.02.005 23474085

[B28] Nicholson J. K. Holmes E. Kinross J. Burcelin R. Gibson G. Jia W. (2012). Host-gut microbiota metabolic interactions. *Science* 336 1262–1267. 10.1126/science.1223813 22674330

[B29] Noguchi H. Park J. Takagi T. (2006). MetaGene: prokaryotic gene finding from environmental genome shotgun sequences. *Nucleic Acids Res.* 34 5623–5630. 10.1093/nar/gkl723 17028096 PMC1636498

[B30] Patel D. D. Patel A. K. Parmar N. R. Shah T. M. Patel J. B. Pandya P. R. (2014). Microbial and Carbohydrate Active Enzyme profile of buffalo rumen metagenome and their alteration in response to variation in the diet. *Gene* 545 88–94. 10.1016/j.gene.2014.05.003 24797613

[B31] Pedersen R. Ingerslev H. Sturek M. Alloosh M. Cirera S. Christoffersen B. O. (2013). Characterisation of gut microbiota in Ossabaw and Gottingen minipigs as models of obesity and metabolic syndrome. *PLoS ONE* 8:e56612 10.1371/journal.pone.0056612 PMC3577853 23437186

[B32] Qin J. Li R. Raes J. Arumugam M. Burgdorf K. S. Manichanh C. (2010). A human gut microbial gene catalogue established by metagenomic sequencing. *Nature* 464 59–65. 10.1038/nature08821 20203603 PMC3779803

[B33] Qin N. Yang F. Li A. Prifti E. Chen Y. Shao L. (2014). Alterations of the human gut microbiome in liver cirrhosis. *Nature* 513 59–64. 10.1038/nature13568 25079328

[B34] Ramayo-Caldas Y. Mach N. Lepage P. Levenez F. Denis C. Lemonnier G. (2016). Phylogenetic network analysis applied to pig gut microbiota identifies an ecosystem structure linked with growth traits. *ISME J.* 10 2973–2977. 10.1038/ismej.2016.77 27177190 PMC5148198

[B35] Ridaura V. K. Faith J. J. Rey F. E. Cheng J. Duncan A. E. Kau A. L. (2013). Gut microbiota from twins discordant for obesity modulate metabolism in mice. *Science* 341 1241214–1241214. 10.1126/science.1241214 24009397 PMC3829625

[B36] Sami M. Yamashita H. Hirono T. Kadokura H. Kitamoto K. Yoda K. (1997). Hop-resistant *Lactobacillus brevis* contains a novel plasmid harboring a multidrug resistance-like gene. *J. Ferment. Bioeng.* 84 1–6. 10.1016/S0922-338X(97)82778-X

[B37] Segata N. Izard J. Waldron L. Gevers D. Miropolsky L. Garrett W. S. (2011). Metagenomic biomarker discovery and explanation. *Genome Biol.* 12:R60 10.1186/gb-2011-12-6-r60 PMC3218848 21702898

[B38] Singh K. M. Shah T. Deshpande S. Jakhesara S. J. Koringa P. G. Rank D. N. (2012). High through put 16S rRNA gene-based pyrosequencing analysis of the fecal microbiota of high FCR and low FCR broiler growers. *Mol. Biol. Rep.* 39 10595–10602. 10.1007/s11033-012-1947-7 23053958

[B39] Singh K. M. Shah T. M. Reddy B. Deshpande S. Rank D. N. Joshi C. G. (2014). Taxonomic and gene-centric metagenomics of the fecal microbiome of low and high feed conversion ratio (FCR) broilers. *J. Appl. Genet.* 55 145–154. 10.1007/s13353-013-0179-4 24136777

[B40] Takada T. Watanabe K. Makino H. Kushiro A. (2016). Reclassification of *Eubacterium desmolans* as *Butyricicoccus desmolans* comb. nov., and description of *Butyricicoccus faecihominis* sp nov., a butyrate-producing bacterium from human faeces. *Int. J. Syst. Evol. Microbiol.* 66 4125–4131. 10.1099/ijsem.0.001323 27453394

[B41] Tremaroli V. Backhed F. (2012). Functional interactions between the gut microbiota and host metabolism. *Nature* 489 242–249. 10.1038/nature11552 22972297

[B42] Turnbaugh P. J. Baeckhed F. Fulton L. Gordon J. I. (2008). Diet-induced obesity is linked to marked but reversible alterations in the mouse distal gut microbiome. *Cell Host Microbe* 3 213–223. 10.1016/j.chom.2008.02.015 18407065 PMC3687783

[B43] Turnbaugh P. J. Ley R. E. Mahowald M. A. Magrini V. Mardis E. R. Gordon J. I. (2006). An obesity-associated gut microbiome with increased capacity for energy harvest. *Nature* 444 1027–1031. 10.1038/nature05414 17183312

[B44] Xiao L. Estelle J. Kiilerich P. Ramayo-Caldas Y. Xia Z. Feng Q. (2016). A reference gene catalogue of the pig gut microbiome. *Nat. Microbiol.* 10.1038/nmicrobiol.2016.161 [Epub ahead of print]. 27643971

[B45] Xing K. Zhu F. Zhai L. Chen S. Tan Z. Sun Y. (2016). Identification of genes for controlling swine adipose deposition by integrating transcriptome, whole-genome resequencing, and quantitative trait loci data. *Sci. Rep.* 6:23219 10.1038/srep23219 PMC4800386 26996612

[B46] Yang H. Huang X. Fang S. Xin W. Huang L. Chen C. (2016). Uncovering the composition of microbial community structure and metagenomics among three gut locations in pigs with distinct fatness. *Sci. Rep.* 6:27427 10.1038/srep27427 PMC4891666 27255518

[B47] Yang L. Bian G. Su Y. Zhu W. (2014). Comparison of faecal microbial community of lantang, bama, erhualian, meishan, xiaomeishan, duroc, landrace, and yorkshire sows. *Asian Australas. J. Anim.* 27 898–906. 10.5713/ajas.2013.13621 PMC4093183 25050029

