# Supplementary figures and images for: Metagenomic Analysis of Cecal Microbiome Identified Microbiota and Functional Capacities Associated with Feed Efficiency in Landrace Finishing Pigs
Source: Front Microbiol. 2017 Aug 11;8:1546. doi: 10.3389/fmicb.2017.01546 (PMC5554500; doi:10.3389/fmicb.2017.01546)

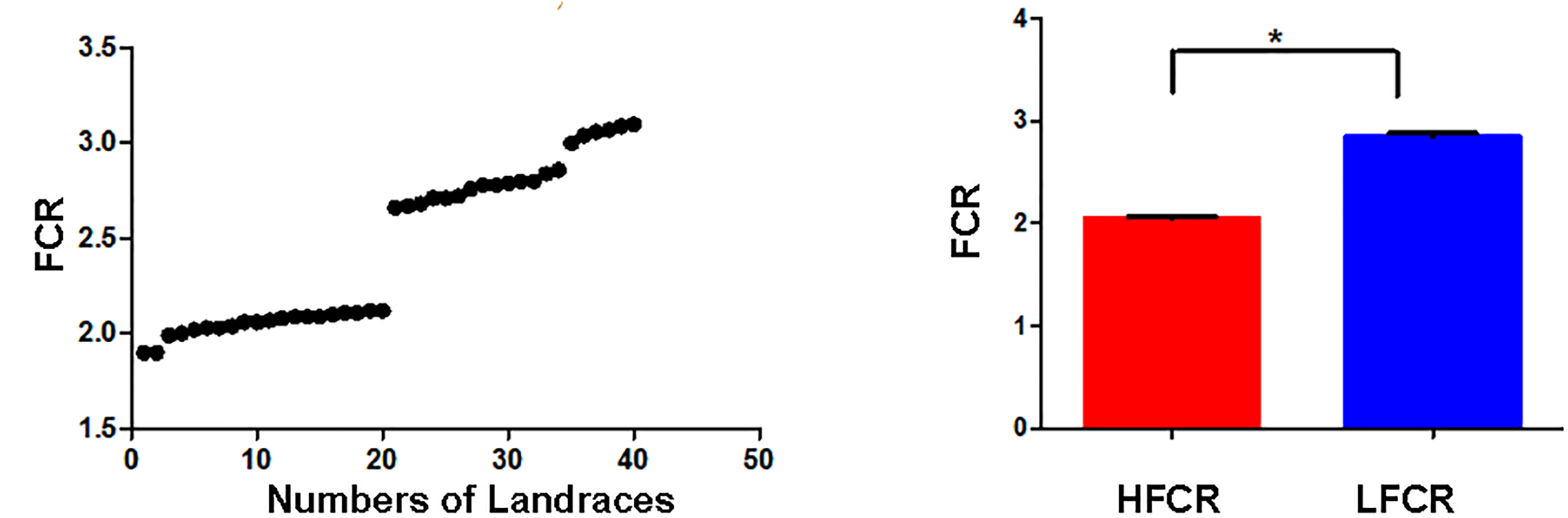

Supplement: FIGURE S1 — Feed Conversion Ratio (FCR) calculated in high and low groups. Significant difference was tested of 20 individuals at the ends of the high and low FCR, respectively, by one way variance analysis. [file Image_1.TIF]

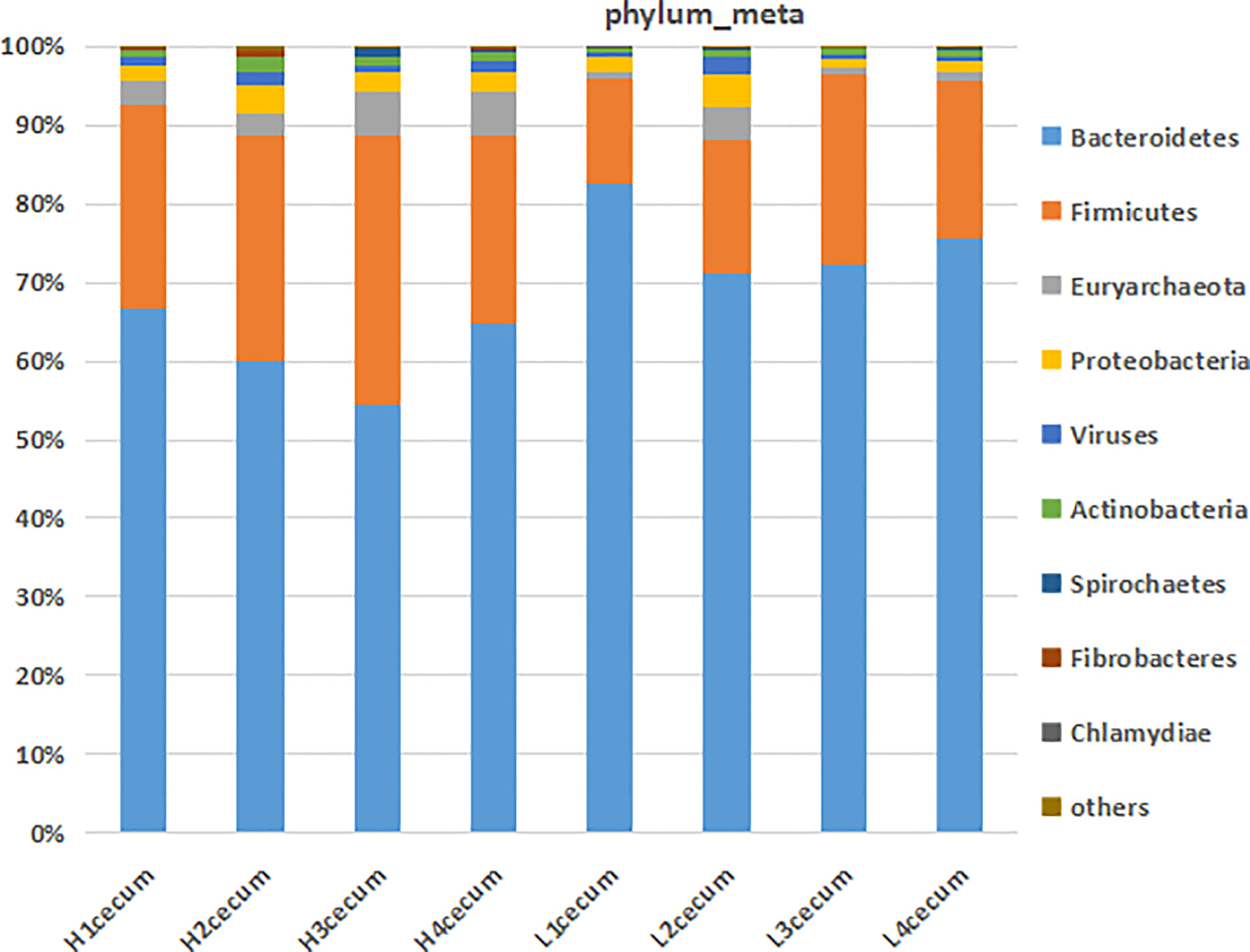

Supplement: FIGURE S2 — Taxonomic composition calculated by different methods at the phylum level based on metagenomics. [file Image_2.TIF]

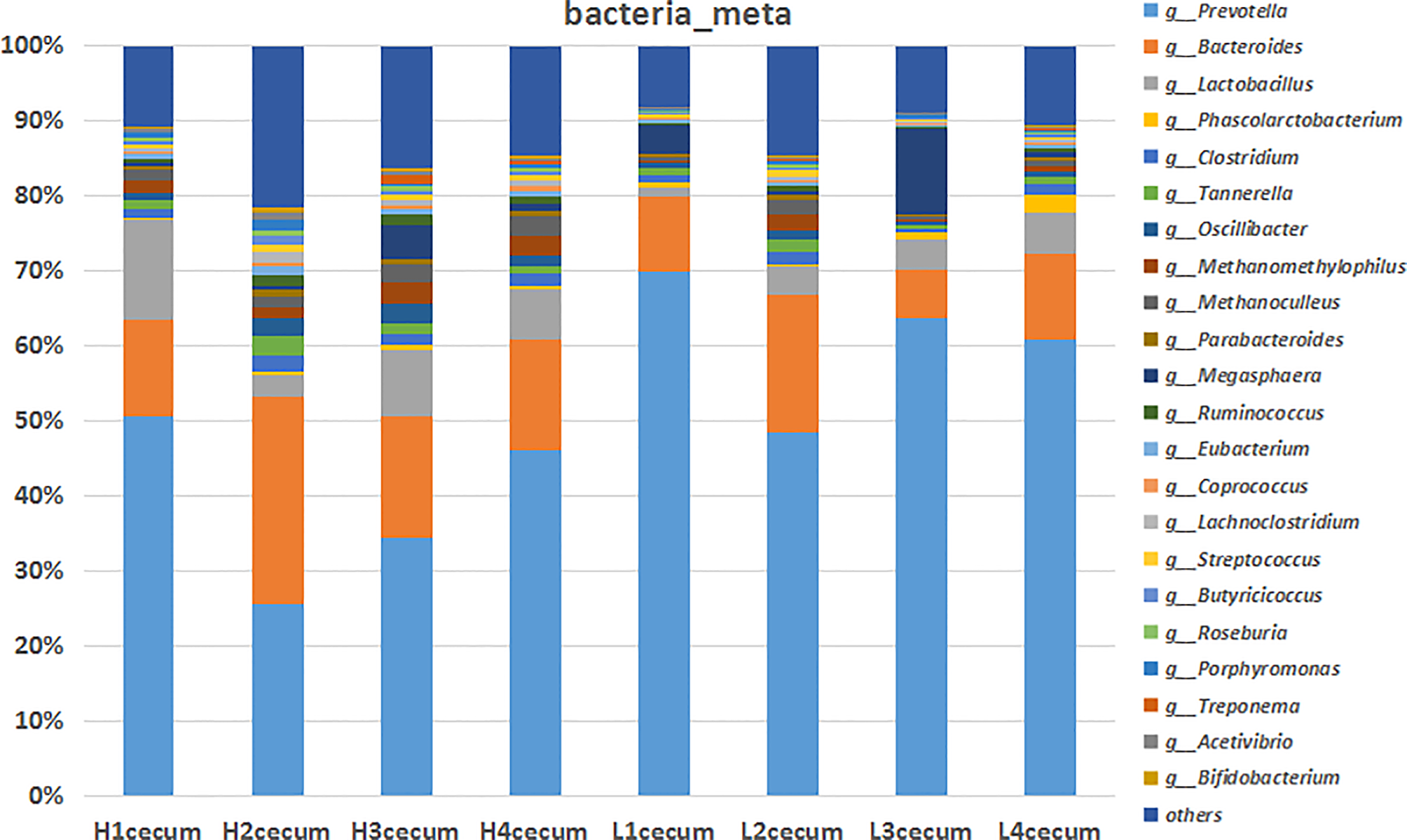

Supplement: FIGURE S3 — Taxonomic composition calculated by different methods at the genus level based on metagenomics. [file Image_3.TIF]

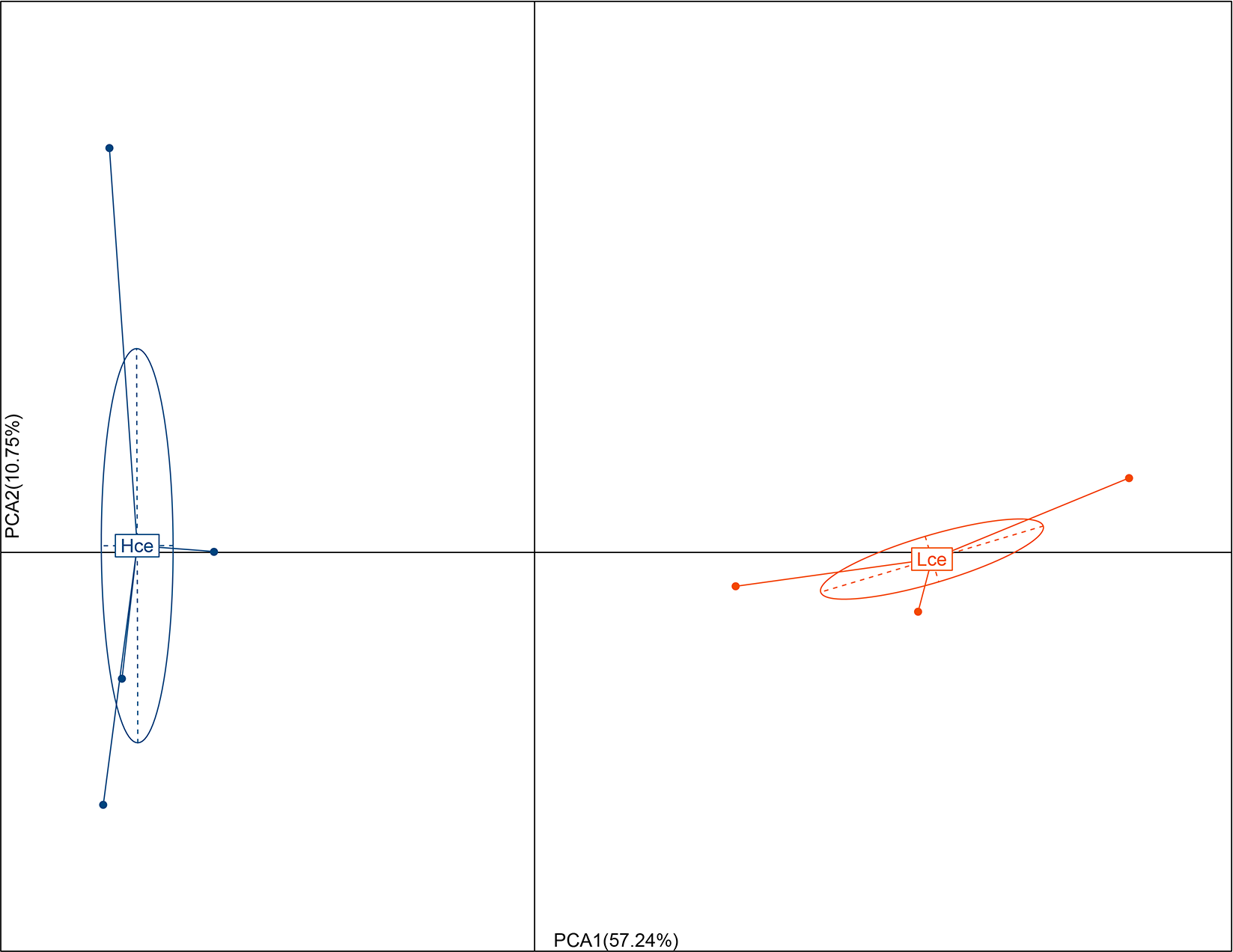

Supplement: FIGURE S4 — principal component analysis (PCA) for significant different predicted genes between the Hce and Lce groups. Hce, cecal predicted genes of high FE group. Lce, cecal predicted genes of low FE group. [file Image_4.TIF]

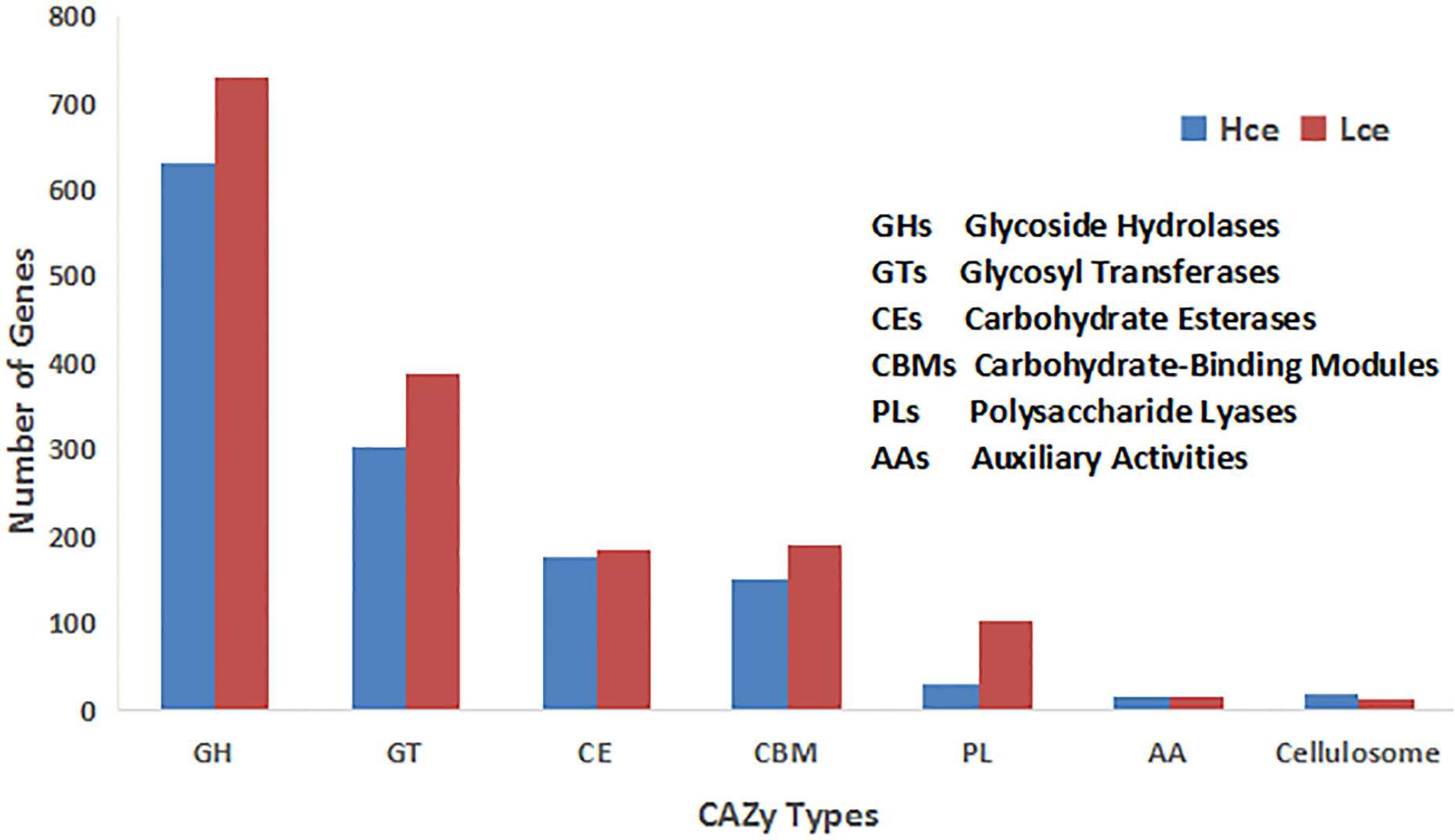

Supplement: FIGURE S5 — Number of different abundant genes clustered by CAZy in Hce and Lce groups. CAZy, Carbohydrate-Active Enzymes Database; Hce, cecal predicted genes of high FE group; Lce, cecal predicted genes of low FE group. [file Image_5.TIF]

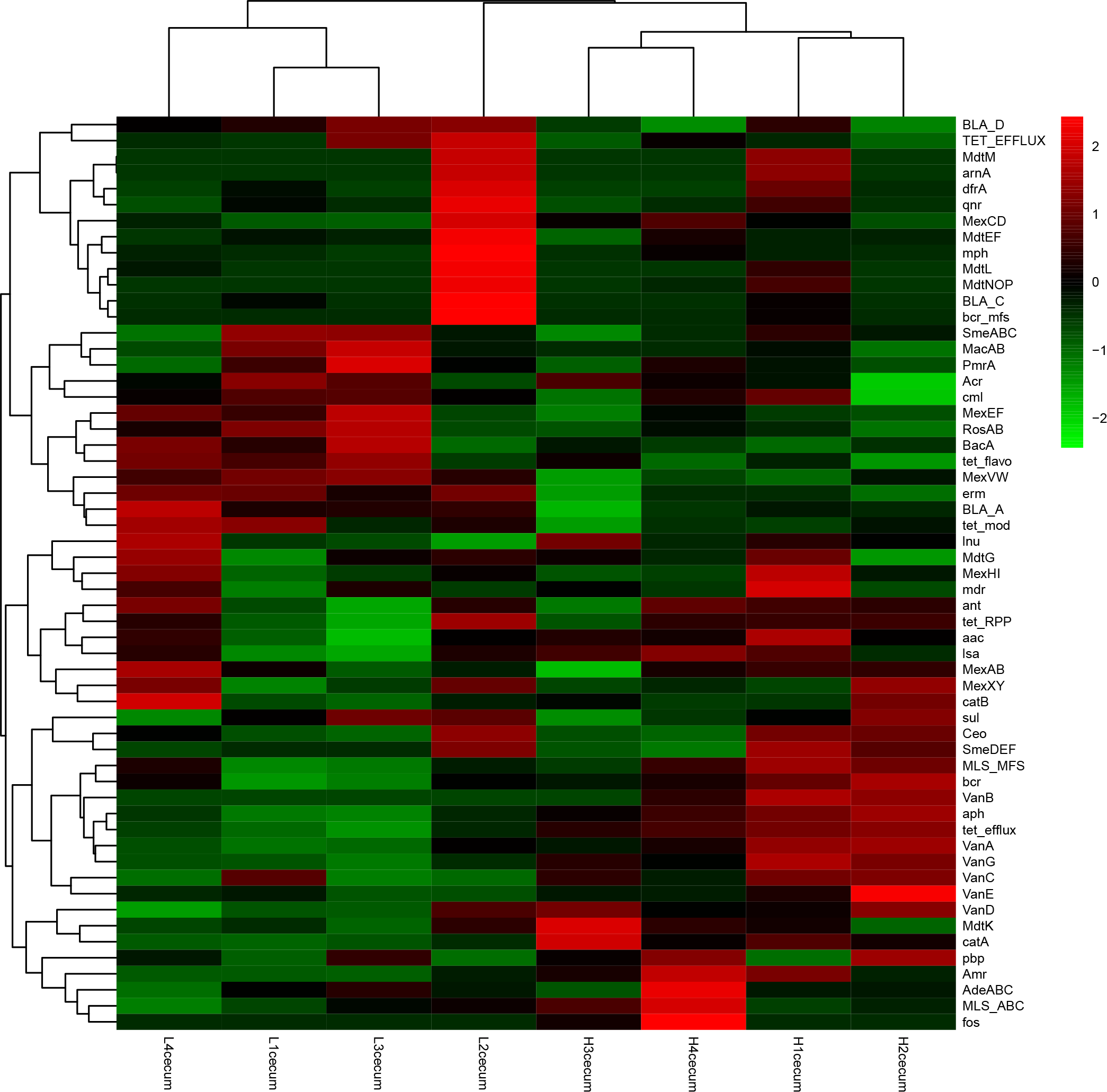

Supplement: FIGURE S6 — Heatmap diagram showing the abundance of antibiotic resistant genes differs between the cecal microbiota of high and low FE groups. FE, feed efficiency. Homogenization control of row by z-score. [file Image_6.TIF]
